# Fast men slow more than fast women in a 10 kilometer road race

**DOI:** 10.7717/peerj.2235

**Published:** 2016-07-21

**Authors:** Robert O. Deaner, Vittorio Addona, Rickey E. Carter, Michael J. Joyner, Sandra K. Hunter

**Affiliations:** 1 Psychology Department, Grand Valley State University, Allendale, MI, United States; 2 Department of Mathematics, Statistics, and Computer Science, Macalester College, Saint Paul, MN, United States; 3 Department of Health Sciences Research, Mayo Clinic, Rochester, MN, United States; 4 Department of Anesthesiology, Mayo Clinic, Rochester, MN, United States; 5 Exercise Science Program, Department of Physical Therapy, Marquette University, Milwaukee, WI, United States

**Keywords:** Gender, Risk taking, Endurance exercise, Distance running, Athletic performance, Marathon, Pacing, Sex differences, Road races, Decision making

## Abstract

**Background.** Previous studies have demonstrated that men are more likely than women to slow in the marathon (footrace). This study investigated whether the sex difference in pacing occurs for a shorter race distance.

**Materials &** **Methods.** Data were acquired from the Bolder Boulder 10 km road race for the years 2008–2013, which encompassed 191,693 performances. There were two pacing measures, percentage change in pace of the first 3 miles relative to the final 3.2 miles and percentage change in pace of the first mile relative to the final 5.2 miles. Pacing was analyzed as a continuous variable and as two categorical variables, as follows: “maintain the pace,” defined as slowing <5% and “marked slowing,” defined as slowing ≥10%.

**Results.** Among the fastest (*men* < 48:40; *women* < 55:27) and second fastest (*men* < 53:54; *women* < 60:28) sex-specific finishing time sextiles, men slowed significantly more than women with both pacing measures, but there were no consistently significant sex differences in pacing among the slower four sextiles. For the fastest sextile, the odds for women were 1.96 (first pacing measure) and 1.36 (second measure) times greater than men to maintain the pace. For the fastest sextile, the odds for women were 0.46 (first measure) and 0.65 (second measure) times that of men to exhibit marked slowing. Multiple regression indicated that being older was associated with lesser slowing, but the sex difference among faster runners persisted when age was controlled.

**Conclusions.** There was a sex difference in pacing during a 10 km race where glycogen depletion is not typically relevant. These results support the hypothesis that the sex difference in pacing partly reflects a sex difference in decision making.

## Introduction

Scientists have long been interested in pacing in endurance events. They have characterized successful pacing trajectories in races of varying lengths and also identified several physiological, energetic, and environmental factors that influence pacing (Abbiss & Laursen, 2008; Tucker & Noakes, 2009; Roelands et al., 2013). Recently, researchers discovered a robust pattern that suggests additional influences on pacing. The pattern is that men are more likely than women to slow their pace in the marathon (42.195 km) (March et al., 2011; Trubee et al., 2014; Deaner et al., 2015a; Krawczyk & Wilamowski, 2015; Hubble & Jinger, 2015; Hanley, 2016). For example, a study of 14 US marathons, including more than 90,000 performances, found that, on average, men ran the second half of the marathon 15.6% slower than the first half, whereas women slowed by 11.7% (Deaner et al., 2015a). Furthermore, the same study showed that men were approximately three times as likely as women to slow by least 30%.

The sex difference in marathon pacing might be attributed to a sex difference in physiology. In particular, it was hypothesized that the sex difference is due to men’s greater susceptibility to muscle glycogen depletion (March et al., 2011; Trubee et al., 2014), a major contributor to marathon slowing (Coyle, 2007; Rapoport, 2010). Supporting this hypothesis are studies suggesting that, for any given intensity of endurance exercise, women are more likely than men to spare glycogen (Roepstorff et al., 2002; Tarnopolsky, 2008).

It was also hypothesized that the sex difference in marathon pacing reflects a sex difference in some aspect of decision making (Deaner et al., 2015a; Krawczyk & Wilamowski, 2015; Hubble & Jinger, 2015). Supporting this hypothesis are recent studies of the Warsaw marathon (Krawczyk & Wilamowski, 2015) and the Houston marathon (Hubble & Jinger, 2015) that examined the relationship between runners’ pacing and the discrepancy between their pre-race self-forecasts and their actual performances; in both marathons, pacing and forecasting discrepancy were substantially correlated, and men’s greater slowing could be largely attributed to their more discrepant forecasting, which may, in turn, be caused by greater over-confidence or risk taking. Also consistent with the decision making hypothesis is the fact that, in non-sport domains, men generally perceive lesser risks than women do (Harris, Jenkins & Glaser, 2006), and laboratory studies indicate that individuals with lesser perceptions of risk are more likely to select ambitious initial paces (Micklewright et al., 2015).

The susceptibility to glycogen depletion hypothesis and the decision making hypothesis are mutually compatible because the decision to adopt an ambitious or risky pace relative to one’s ability will impose physiological challenges. Nevertheless, it could be fruitful to attempt to differentiate these hypotheses in order to clarify the factors that contribute to group and individual variation in pacing. One approach to differentiating these hypotheses is to test for a sex difference in pacing in a distance running event where glycogen depletion should be irrelevant, generally an event shorter than 25 km (Coyle, 2007; Rapoport, 2010). If men’s greater susceptibility to glycogen depletion is the main cause of the sex difference in pacing, then no sex difference should occur in a shorter race. However, if decision making contributes to the sex difference in pacing, then a sex difference, where men slow more than women, should occur in a shorter race.

Here we attempt to differentiate these hypotheses by examining pacing in the Bolder Boulder 10 km road race, which is one of the largest road races in the US (Running USA, 2015) and one of the few large road races shorter than a marathon to record split times. For races occurring between 2008 and 2013, we obtained splits at each mile for 191,693 performances. In addition to testing for an overall sex difference in pacing, we will explore if finishing time and age modulate any sex difference; previous studies of marathon pacing have indicated the importance of these factors (March et al., 2011; Trubee et al., 2014; Santos-Lozano et al., 2014; Deaner et al., 2015a; Krawczyk & Wilamowski, 2015; Hubble & Jinger, 2015; Hanley, 2016).

In summary, the aim of this study is to provide the first test of whether the sex difference in pacing that has been documented for the marathon also occurs in a large 10 km road race. The decision making hypothesis predicts there will be a sex difference in pacing whereas the glycogen depletion hypothesis predicts there will not be a sex difference.

## Materials & Methods

This study did not require formal approval by Grand Valley State’s institutional review board (IRB) or the other IRBs (Macalester College, Marquette University, Mayo Clinic). The Grand Valley State University IRB determined that the protocol (reference number 756083-1) was exempt under federal category 45 CFR 46.101(b) (4) because all data were preexisting and public.

Results from the Bolder Boulder can be publicly accessed (http://onlineraceresults.com/search/ with keyword “Bolder”). Information is available on finishers’ names, hometowns, sex, age, and finishing times for races beginning in 1980; beginning with the 2008 race, mile splits are also available. To speed data acquisition, we requested that all data be sent to us as a single file. The race organizers and timing company generously agreed to this request, although they first removed finishers’ names. The data set spanned 6 years (2008–2013) and initially included all finishers who had sex, age, finishing time, and all splits recorded (*n* = 274,966). We excluded all finishers which we believe had one or more data entry errors, according to the following rules: (i) one or more mile times less than 2 min 30 s or (ii) mile times not consistent with their total finishing time (*n* = 9).

We also excluded all those who finished in 83 min 20 s or greater (*n* = 83,264; 67.76% women). We did this because the transition speed between walking and running typically occurs around 2.0 m/s (or 4.5 mph), so we assumed that runners whose overall speed was slower than this (i.e., a finishing time ≥ 83 min and 20 s) were likely walking a substantial portion of the race. Although there is individual variability in the transition speed between walking and running, this variation appears unrelated to oxygen consumption (Rotstein et al., 2005; Monteiro et al., 2011). Thus, we used this same exclusion criterion for men and women of all ages. After making these exclusions, the sample was comprised of 191,693 usable performances, including 48.85% women (*n* = 93,643).

### Statistical analysis

We considered two measures of pacing. For our first pacing measure, we calculated the percentage change in the pace maintained over the first 3 miles relative to the final 3.2 miles. That is, % change = (minutes per mile in final 3.2 miles − minutes per mile in first 3 miles)/minutes per mile in first 3 miles. We used this measure because it is similar to comparing the pace of the first half of the race to the second half of the race, which has been done in marathon studies (Deaner et al., 2015a; Krawczyk & Wilamowski, 2015). (No 5 km split was available for this race, preventing a more direct comparison with previous studies.) Our second pacing measure was the percentage change in the pace of the first mile relative to the final 5.2 miles. That is, % change = (minutes per mile in final 5.2 miles − minutes per mile in first mile)/minutes per mile in first mile. We selected this measure because it should capture runners beginning the race at a speed which is too fast to be sustainable.

We considered each of these two pacing measures as a continuous variable and as two categorical variables. For the first categorization, percentage changes less than 5% were considered “maintaining the pace.” For example, a runner who completed the first 3 miles of the race in 6 min and 40 s per mile and the final 3.2 miles in 7 min per mile or better maintained the pace; those who ran the final 3.2 miles in more than 7 min per mile failed to maintain the pace. For the second categorization, percentage changes equal to or greater than 10% were considered as “marked slowing.” For example, a runner who completed the first 3 miles of the race in 6 min and 40 s per mile and the final 3.2 miles in 7 min and 20 s per mile or worse showed marked slowing. Total finishing time and all mile split times were determined from electronic chip times (i.e., based on when each individual crossed the starting line).

To explore the impact of finishing time on our categorical pacing measures, we also categorized male and female performances into sex-specific sextiles (i.e., dividing all performances of one sex into six equivalent sized groups). The fastest male group consisted of men finishing in 48:40 or faster, and the other sextile boundaries for men were 53:54, 58:30, 63:40, and 71:05. The corresponding boundaries for women were 55:27, 60:28, 64:58, 69:53, and 75:41.

When considering pacing as a continuous response (or dependent) variable, we estimated two multiple regression models for each of our two pacing measures. Model 1 used sex, age, and finishing time as explanatory (or independent) variables. Model 2 included sex, age, and finishing time, along with the pairwise interactions between these variables. For these models and some figures, we followed a previous study (Deaner et al., 2015a) in dividing women’ times by 1.12 to account for men’s greater maximal oxygen uptake (Joyner, 1993; Sparling, O’Donnell & Snow, 1998; Cheuvront et al., 2005).

Descriptive summaries are presented as mean ± standard deviation (SD). All analyses were conducted using the R programming language, version 3.1.1 (R Core Team, 0000). Data are reported as mean ± SD in the text. Two-sided *P*-values less than 0.05 were taken as statistically significant.

## Results

A total of 191,693 runners (48.85% women) were included in this analysis. The ages for men and women, respectively, were 35.54 ± 15.27 and 32.90 ± 12.41 years. Finishing times for men and women were 59:23 ± 10:51 and 65:10 ± 9:36 min, respectively, a difference in means of 8.9%.

The percentage changes in pace of the first 3 miles relative to the final 3.2 miles were 2.02 ± 6.69 and 1.71 ± 5.90% for men and women, respectively (*P* < 0.0001). The percentage changes in pace of the first mile relative to the final 5.2 miles were 8.17 ± 9.38 and 8.04 ± 8.62% for men and women, respectively (*P* = 0.0024).

### Pacing by sex and finishing time

Table 1 reports the results for the two pacing measures (Measure 1: first 3 miles relative to the final 3.2 miles; Measure 2: first mile relative to the final 5.2 miles) for each finishing time sextile, separately for men and women. For both measures and both sexes, change in pace varied significantly across sextiles (*P* < 0.0001 for both sexes and both measures), with slower runners generally experiencing greater slowing. Among runners in the fastest two sextiles, men slowed significantly more than women for both pacing measures. Among the other sextiles, women sometimes slowed significantly more than men (fourth sextile, measures 1 and 2; fifth sextile, measure 2), men sometimes slowed significantly more than women (third sextile, measure 1; sixth sextile, measures 1 and 2), and in some cases, there was no significant sex difference (fifth sextile, measure 1; third sextile, measure 2).

**Table 1:** Pacing by sex and finishing time sextile. Pacing Measure 1 indicates percentage change in pace of the first 3 miles relative to the final 3.2 miles. Pacing Measure 2 indicates percentage change in pace of the first mile relative to the final 5.2 miles.

Sextile (# of Women, # of Men)	Pacing measure 1			Pacing measure 2		
	Women	Men	*P*-value	Women	Men	*P*-value
	Mean (SD)	Mean (SD)		Mean (SD)	Mean (SD)	
Fastest sextile (15,649, 16,344)	0.62 (3.61)	1.62 (3.88)	<0.0001	4.82 (5.39)	6.08 (5.77)	<0.0001
Second sextile (15,607, 16,352)	0.46 (4.50)	1.17 (4.97)	<0.0001	4.91 (6.41)	5.84 (7.12)	<0.0001
Third sextile (15,585, 16,334)	1.09 (5.09)	1.25 (5.76)	0.0071	6.04 (7.10)	6.14 (8.04)	0.23
Fourth sextile (15,606, 16,342)	1.96 (5.89)	1.48 (6.56)	<0.0001	8.05 (8.02)	7.01 (9.00)	<0.0001
Fifth sextile (15,608, 16,341)	2.79 (6.85)	2.75 (7.56)	0.62	10.69 (9.35)	9.72 (9.99)	<0.0001
Slowest sextile (15,588, 16,337)	3.35 (7.83)	3.83 (9.48)	<0.0001	13.78 (10.47)	14.24 (12.00)	0.00027

We next determined, for each pacing measure, the percentage of men and women in each sextile that maintained the pace (<5% slowing). Table 2 shows that, for both pacing measures, maintaining the pace varied across sextiles, as faster runners were more likely than slower runners to maintain the pace (*P* < 0.0001 for both sexes and both measures).

**Table 2:** Percentage of runners who maintained the pace (<5% slowing) by sex and finishing time sextile. Pacing Measure 1 indicates percentage change in pace of the first 3 miles relative to the final 3.2 miles. Pacing Measure 2 indicates percentage change in pace of the first mile relative to the final 5.2 miles. The odds ratio (OR) compares the odds of maintaining the pace for women compared with men.

Sextile (# of Women, # of Men)	Pacing Measure 1			Pacing Measure 2		
	Women	Men	OR (95% CI)	Women	Men	OR (95% CI)
	% maintained pace	% maintained pace		% maintained pace	% maintained pace	
Fastest sextile (15,649, 16,344)	91.29	84.28	1.96 (1.82,2.10)	52.43	44.78	1.36 (1.30,1.42)
Second sextile (15,607, 16,352)	87.22	82.29	1.47 (1.38,1.56)	51.49	46.46	1.22 (1.17,1.28)
Third sextile (15,585, 16,334)	81.07	78.64	1.16 (1.10,1.23)	45.17	45.40	0.99 (0.95,1.04)
Fourth sextile (15,606, 16,342)	73.27	74.45	0.94 (0.90,0.99)	35.47	42.71	0.74 (0.71,0.77)
Fifth sextile (15,608, 16,341)	65.91	65.85	1.00 (0.96,1.05)	24.72	32.08	0.70 (0.66,0.73)
Slowest sextile (15,588, 16,337)	61.96	59.17	1.12 (1.08,1.18)	16.37	18.74	0.85 (0.80,0.90)
Overall (93,643, 98,050)	76.80	74.12	1.16 (1.13,1.18)	37.62	38.36	0.97 (0.95,0.99)

We calculated the common (pooled) OR for maintaining the pace for women compared with men across the sextiles (Table 2). Overall, the odds that women maintained the pace were 1.16 times (95% CI [1.13–1.18]; *P* < 0.0001) greater compared to men for the first pacing measure. For the second pacing measure, the odds that women maintained the pace were 0.97 times as great as the odds that men maintained the pace (95% CI [0.95–0.99]; *P* < 0.0001). For both measures, there were significant differences among the sextiles (*P* < 0.0001 for both measures, indicated by a Breslow–Day test for homogeneity). The largest sex differences occurred in the fastest sextile (first measure: OR, 1.96; 95% CI [1.82–2.10]; second measure: OR, 1.36; 95% CI [1.30–1.42]).

We conducted similar analyses of marked slowing (≥10% slowing). Table 3 shows that, for both measures, marked slowing varied across sextiles, as faster runners were less likely to exhibit marked slowing (*P* < 0.0001 for both sexes). Overall, the odds of exhibiting marked slowing were 0.79 (95% CI [0.76–0.81]; *P* < 0.0001) times as great for women compared with men for the first pacing measure, and 0.98 times (95% CI [0.97–1.00]; *P* = 0.10) as great for women compared with men for the second pacing measure (Table 3). There were significant differences among the sextiles (*P* < 0.0001 for both measures). The greatest sex differences occurred in the fastest sextile (first measure: OR, 0.46; 95% CI [0.38–0.55]; second measure: OR, 0.65; 95% CI [0.61–0.68]).

**Table 3:** Percentage of runners who exhibited marked slowing (≥10% slowing) by sex and finishing time sextile. Pacing Measure 1 indicates percentage change in pace of the first 3 miles relative to the final 3.2 miles. Pacing Measure 2 indicates percentage change in pace of the first mile relative to the final 5.2 miles. The odds ratio (OR) compares the odds of experiencing marked slowing for women compared with men.

Sextile (# of Women, # of Men)	Pacing measure 1			Pacing measure 2		
	Women	Men	OR (95% CI)	Women	Men	OR(95% CI)
	% marked slowing	% marked slowing		% marked slowing	% marked slowing	
Fastest sextile (15,649, 16,344)	0.97	2.10	0.46 (0.38,0.55)	15.37	21.97	0.65 (0.61,0.68)
Second sextile (15,607, 16,352)	1.97	3.27	0.59 (0.52,0.68)	19.39	24.74	0.73 (0.69,0.77)
Third sextile (15,585, 16,334)	3.70	5.19	0.70 (0.63,0.78)	26.08	27.89	0.91 (0.87,0.96)
Fourth sextile (15,606, 16,342)	7.11	7.39	0.96 (0.88,1.05)	36.82	32.52	1.21 (1.16,1.27)
Fifth sextile (15,608, 16,341)	11.15	13.29	0.82 (0.77,0.88)	50.95	45.15	1.26 (1.21,1.32)
Slowest sextile (15,588, 16,337)	15.04	18.69	0.77 (0.73,0.82)	65.23	63.67	1.07 (1.02,1.12)
Overall (93,643, 98,050)	6.65	8.32	0.79 (0.76,0.81)	35.63	35.99	0.98 (0.97,1.00)

**Figure 1:**
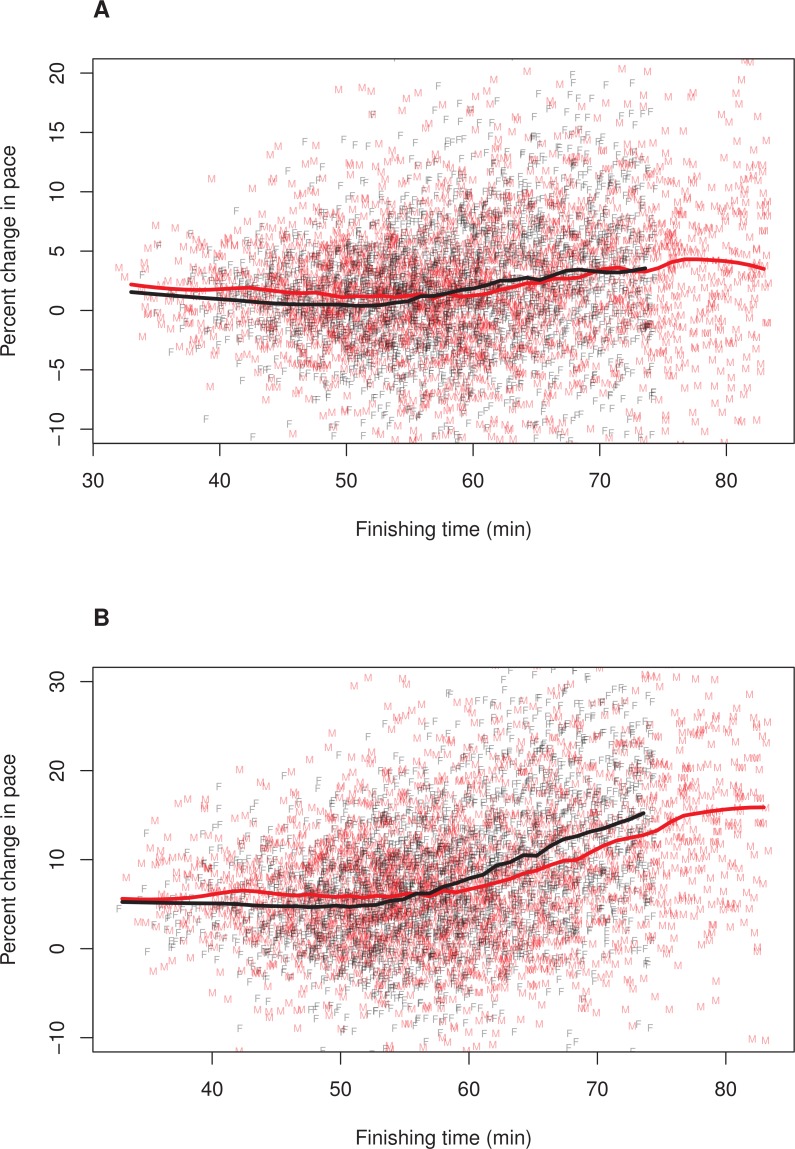
Pacing as a function of finishing time for men and women. A random subset of 5,000 data points are plotted, along with a loess smoother (obtained using all of the data) separately for men (red) and women (black). Women’s finishing times presented on the *x*-axis have been adjusted (i.e., decreased) by 12%. (A) Pacing Measure 1, percentage change in pace of the first 3 miles relative to the final 3.2 miles. (B) Pacing Measure 2, percentage change in pace of the first mile relative to the final 5.2 miles.

**Figure 2:**
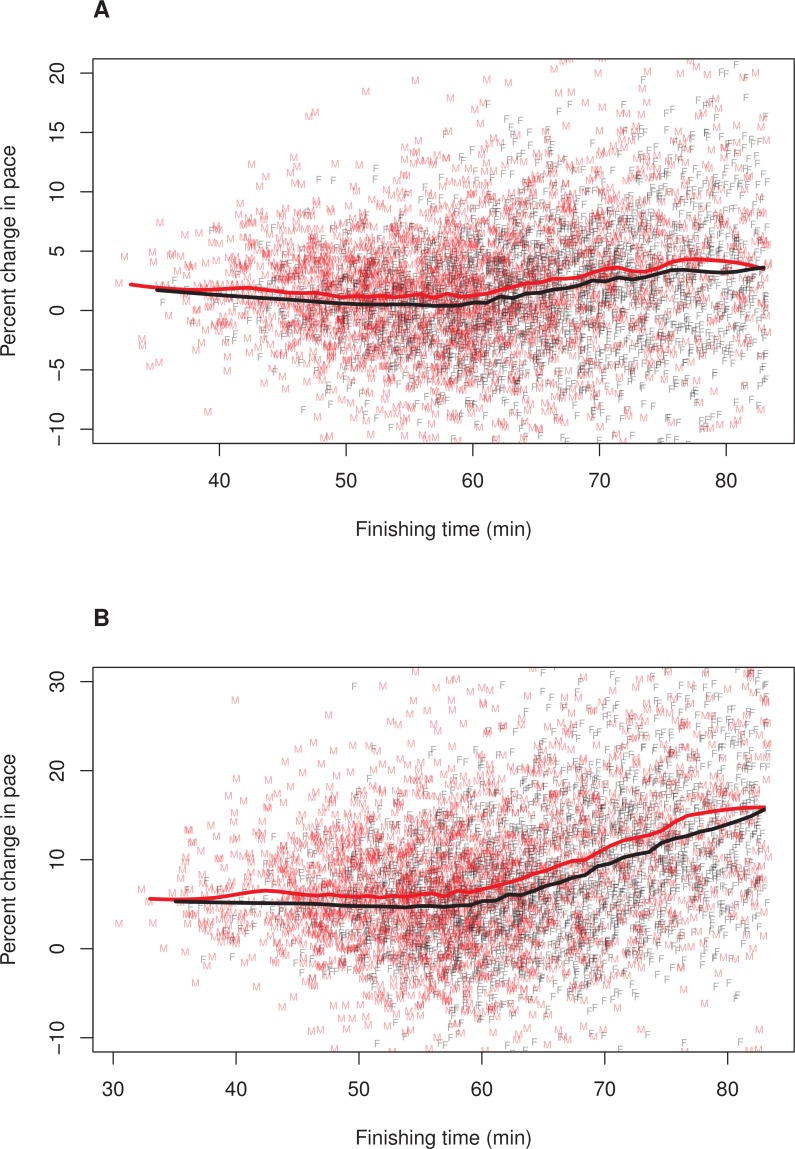
Pacing as a function of finishing time for men and women without 12% adjustment to women’s times. A random subset of 5,000 data points are plotted, along with a loess smoother (obtained using all of the data) separately for men (red) and women (black). Women’s finishing times presented on the *x*-axis have not been adjusted by 12%. (A) Pacing Measure 1, percentage change in pace of the first 3 miles relative to the final 3.2 miles. (B) Pacing Measure 2, percentage change in pace of the first mile relative to the final 5.2 miles

To further explore whether the relationship between finishing time and pacing differed for men and women, we plotted these relationships separately for each sex. In these plots, we adjusted (i.e., decreased) female finishing times by 12% (see ‘Methods’). Figure 1 illustrates the results, (A) Pacing Measure 1, and (B) Pacing Measure 2. This figure indicates that, for both measures, slower finishers exhibited greater percentage slowing, although this trend was most prominent for those who finished in approximately 60 min or more. In addition, this figure shows that, for both measures, men tended to slow more than women among the fastest runners (i.e., roughly those who finished in under 53 min, corresponding to a time of 59:36 for women). Among slower runners, there was no consistent sex difference in pacing for the first pacing measure (Fig. 1A). For the second pacing measure, among slower runners, women showed a consistent tendency to slow more than men (Fig. 1B). We also plotted these relationships without making a 12% adjustment to women’ finishing times (Fig. 2). With these absolute finishing times, men slowed more than women among faster and slower runners, and this was true for both pacing measures.

### Regression modeling to control for runner’s age

The preceding results investigated the relationships among pacing, sex and finishing time through tabular and visual displays. In order to simultaneously address the potential impact of runner’s age, we used multiple regression modeling. We fit two models for each of our two continuous pacing measures. Model 1 used sex, age, and finishing time as explanatory variables. Model 2 additionally included the pairwise interactions between sex, age, and finishing time. In these models, finishing time was measured in minutes (adjusted by 12% for women) and age was measured in years.

For Pacing Measure 1, older, faster, and female runners exhibited more even pacing (Model 1). With regards to sex differences, Model 2 indicated that the effect of sex was attenuated for slower finishers. That is, men generally showed greater slowing than women, but this difference weakened as finishing time increased.

For Pacing Measure 2, older and faster runners showed more even pacing, while males and females were not significantly different (Model 1). The lack of significance of the sex variable in Model 1 reflects a significant interaction between sex and finishing time (Model 2). That is, among fast runners, men showed greater slowing but, among slower finishers, women showed greater slowing.

These regression results corroborated the patterns illustrated in Fig. 1. Complete information on the value of the coefficients and standard errors from these models are shown in Table 4.

**Table 4:** Summary of regression models of pacing (coefficients, with standard errors in parentheses). Pacing measure 1 indicates percentage change in pace of the first 3 miles relative to the final 3.2 miles. Pacing measure 2 indicates percentage change in pace of the first mile relative to the final 5.2 miles. See text for descriptions of Model 1 and Model 2.

Explanatory variable	Pacing measure 1		Pacing measure 2	
	Model 1	Model 2	Model 1	Model 2
(Intercept)	−3.37 (0.092)^***^	−3.99 (0.25)^***^	−6.89 (0.13)^***^	−13.48 (0.34)^***^
Sex male	0.21 (0.029)^***^	2.76 (0.19)^***^	−0.029 (0.039)	5.42 (0.26)^***^
Finishing time (adjusted)	0.092 (0.0015)^***^	0.10 (0.0041)^***^	0.30 (0.002)^***^	0.42 (0.0055)^***^
Age	−0.0073 (0.001)^***^	−0.038 (0.0063)^***^	−0.078 (0.0014)^***^	0.0044 (0.0086)
Sex male: finishing time	–	−0.045 (0.0030)^***^	–	−0.11 (0.0041)^***^
Sex male: age	–	0.0030 (0.0021)	–	0.021 (0.0029)^***^
Finishing time: age	–	0.00048 (0.0001)^***^	–	−0.0015 (0.0001)^***^

**Notes.**

*** *p*-value < 0.0001.

## Discussion

This study demonstrated that in the Bolder Boulder race, a large 10 km road race, there was a sex difference in pacing, specifically among faster runners. In particular, men and women in the fastest two sex-specific finishing time sextiles differed significantly in their pacing, with men generally slowing more than women. This was true whether pacing was defined as the change in pace of the first 3 miles relative to the final 3.2 miles (first pacing measure) or as the change in pace of the first mile relative to the final 5.2 miles (second measure). Moreover, categorical comparisons indicated that the magnitude of the sex difference was not trivial (Tables 2 and 3). Perhaps most notably, among the fastest sextile, the odds of women slowing by at least 10% was 0.46 (first pacing measure) and 0.65 (second pacing measure) times that of the corresponding value for men (Table 3). The sex difference in pacing among faster runners also occurred when comparisons were based on continuous finishing times, rather than sex-specific finishing time sextiles, including when female finishing times were adjusted by 12% to account for men’ greater maximal oxygen uptake (Joyner, 1993; Sparling, O’Donnell & Snow, 1998; Cheuvront et al., 2005). Finally, the sex difference in pacing persisted in multiple regression models that controlled for age (Table 4).

Although these results indicate that the sex difference in pacing that has been documented in the marathon (March et al., 2011; Trubee et al., 2014; Deaner et al., 2015a; Krawczyk & Wilamowski, 2015; Hubble & Jinger, 2015; Hanley, 2016) also occurs in shorter race distances, there were some notable differences between pacing at these distances. First, the magnitude of slowing in the present 10 km race was substantially less than has been found in marathons. In this 10 km race, the mean slowing for the first 3 miles relative to the final 3.2 miles was 2.0% for women and 1.7% for men, whereas, in a sample of 14 marathons, the mean slowing for the first half of the marathon relative to the second half was 11.7% for women and 15.6% for men (Deaner et al., 2015a). A second important difference is that, in this 10 km race, the pattern of men slowing more than women was limited to relatively fast runners (Tables 1–3 and Fig. 1). Among slower runners there was no consistent sex difference and, in some analyses, women slowed significantly more than men did. In the 14 marathons, by contrast, men slowed substantially more than women among faster runners, and this sex difference became larger among slower runners (Deaner et al., 2015a). Third, among faster runners, men were consistently more likely than women to slow in this 10 km race, but the magnitude of this sex difference was less than that reported in the 14 marathons. Although ability groupings and categorical definitions of slowing differed in these studies, it is reasonable to conclude there is a difference because odds ratios indicating sex differences were consistently smaller in this 10 km race, even for faster runners, (Tables 2 and 3) than reported in the 14 marathons (Deaner et al., 2015a).

### Susceptibility to glycogen depletion hypothesis

The documentation of a sex difference in pacing in a 10 km road race challenges the hypothesis that the sex difference in pacing is due to men’s greater susceptibility to glycogen depletion (March et al., 2011; Trubee et al., 2014). According to that hypothesis, no sex difference in pacing should have occurred for a 10 km race because glycogen depletion is highly unlikely to be relevant for events of this duration (Coyle, 2007; Rapoport, 2010). Nevertheless, it is premature to completely reject the susceptibility to glycogen depletion hypothesis because the present study of a 10 km race cannot discount that men’s apparently greater susceptibility to glycogen depletion contributes to the sex difference in pacing in marathons. The fact that the sex difference in pacing in this 10 km race was limited to faster runners and was smaller in magnitude than the sex difference in marathon pacing is consistent with this possibility.

### Decision making hypothesis

The present study’s results support, albeit indirectly, the hypothesis that the sex difference in pacing in distance running reflects, at least in part, some kind of sex difference in decision making (Allen & Dechow, 2013; Deaner et al., 2015a; Krawczyk & Wilamowski, 2015; Hubble & Jinger, 2015). For example, men may be more likely than women to begin a race with an ambitious or risky pace relative to their ability, and this would increase their likelihood of slowing later.

The decision making hypothesis is supported by several lines of evidence besides the present study’s results. First, men’s greater slowing in the Warsaw (Krawczyk & Wilamowski, 2015) and Houston marathons (Hubble & Jinger, 2015) was substantially associated with their more discrepant forecasting, indicating men were typically more ambitious or over-confident than were women. Second, a study of the Chicago marathon showed that performances among non-elite runners clustered at round numbers (e.g., just under 4 h) because some runners accelerated in the final 2.2 km of the race; although women were more likely than men to have accelerated in the final 2.2 km, men were more likely to have done so in order to achieve round numbers, indicating that the men were more likely to modify their pace to pursue external goals (Allen & Dechow, 2013). Third, in non-sport domains, men generally take greater risks, partly due to their lesser perceptions of risk (Harris, Jenkins & Glaser, 2006), and laboratory studies of running and cycling indicate that individuals with lesser perceptions of risk are more likely to adopt ambitious initial paces (Micklewright et al., 2015). Finally, risk taking and competitiveness are believed to be closely related (Croson & Gneezy, 2009), and there is mounting evidence that male distance runners are typically more competitive than their female counterparts (Deaner, 2013; Deaner, Addona & Mead, 2014; Deaner et al., 2015b).

Other physiological factor(s) besides glycogen depletion may contribute to the sex difference in pacing. The only suggestion of which we are aware is that the sex difference in pacing might be due to men’s (supposedly) greater susceptibility to hyperthermia (Trubee et al., 2014), a frequent contributor to slowing in distance running (Maughan, Watson & Shirreffs, 2007; Gonzalez-Alonso, Crandall & Johnson, 2008; El Helou et al., 2012). This hypothesis was suggested by the fact that the sex difference in marathon pacing increases with warmer ambient temperatures (Trubee et al., 2014; Krawczyk & Wilamowski, 2015). The present study’s results from the Bolder Boulder 10 km race do not address the susceptibility to hyperthermia hypothesis. (We did not address the effect of temperature because there was little variation in the race years we studied; the maximum daily temperature in Boulder on race day was 23.06 ± 3.31 °C.) We note, however, that this hypothesis is weakened by the fact that, compared to women, men are generally believed to have advantages in thermoregulation (Gagnon & Kenny, 2012). Moreover, the sex difference in pacing has been documented in marathons (March et al., 2011; Trubee et al., 2014; Krawczyk & Wilamowski, 2015) that occurred at temperatures sufficiently cool (e.g., <10 °C) that hyperthermia would have been irrelevant (El Helou et al., 2012).

Another crucial point is that although there is, at present, no compelling evidence that a physiological difference between men and women can explain the sex difference in pacing, the decision making hypothesis is not at odds with physiology contributing to individual and group variation in pacing. This is because the decision to adopt an ambitious or risky pace will undoubtedly impose physiological challenges (Coyle, 2007; Abbiss & Laursen, 2008; Tucker & Noakes, 2009).

### Why men’s greater slowing was limited to faster runners

An interesting question is why the greater slowing by men than women in this 10 km road race was limited to relatively fast runners, roughly men who finished in less than 53 min and women who finished in less than 60 min. By contrast, the sex difference in marathon pacing occurs for runners of widely ranging abilities (March et al., 2011; Trubee et al., 2014; Deaner et al., 2015a; Krawczyk & Wilamowski, 2015). Furthermore, research on sex differences in risk taking in non-sports domains (e.g., driving) generally indicate that sex differences occur across broad populations (Harris, Jenkins & Glaser, 2006; Croson & Gneezy, 2009) or, if they vary across populations, the sex differences tend to be smaller or non-existent among more selective sub-populations (Croson & Gneezy, 2009 but see Deaner et al., 2015b). The sex difference in financial risk taking, for instance, apparently weakens or disappears among professional investors (Croson & Gneezy, 2009).

Although we cannot definitively explain why the sex difference in pacing was limited to relatively fast runners, we can offer a suggestion, based on two points. First, although pacing in distance running can involve risk taking, it often may not. For instance, an evenly paced performance may not indicate a runner whose gamble with an ambitious risky pace paid off; it may simply indicate a runner who adopted a pace that was easy for them. Conversely, a performance with dramatic slowing may not indicate a runner who began the race with an ambitious pace but, despite great effort, failed to hold it; it may merely indicate a runner who did not prioritize achieving an outstanding performance and was content to slow when their initial pace became uncomfortable. Second, although any particular running performance may or may not involve substantial risk taking, it can be expected that faster runners generally will be more likely than slower runners to select an ambitious pace and to exert themselves in maintaining it. This is supported by surveys showing that faster runners are more likely than are slower runners to endorse competitiveness and goal achievement items (e.g., “to push myself beyond my current limits”) (Masters, Ogles & Jolton, 1993; Deaner et al., 2015b). If these points are correct, then variation in pacing among fast runners may provide important insights about risk taking, whereas variation in pacing among slower runners may be less informative.

Our suggestion for why the sex difference in pacing was limited to faster runners in this 10 km race does raise the question of why the sex difference in pacing in marathons held even among slower runners (March et al., 2011; Trubee et al., 2014; Deaner et al., 2015a; Krawczyk & Wilamowski, 2015). Although we cannot provide a definitive answer to this question, we can suggest that 10 km races and marathons may differ in ways that make marathon races more likely to reveal individual and group variation in decision making. One difference is that somewhat different kinds of individuals may participate; marathoners tend to be more motivated by competition (Ogles, Masters & Richardson, 1995). A second difference involves the demands of maintaining an even pace, a reliable correlate of performance in distance running (Abbiss & Laursen, 2008; Tucker & Noakes, 2009; March et al., 2011; Trubee et al., 2014; Santos-Lozano et al., 2014; Deaner et al., 2015a; Krawczyk & Wilamowski, 2015; Hubble & Jinger, 2015; Hanley, 2016). Because fatigue in a marathon generally reflects slowly cumulating processes, such as glycogen depletion (Coyle, 2007; Rapoport, 2010), hyperthermia (Maughan, Watson & Shirreffs, 2007; Gonzalez-Alonso, Crandall & Johnson, 2008; El Helou et al., 2012), and muscle damage (Del Coso et al., 2013), coaching handbooks advise that achieving an evenly paced marathon requires running the first half of the race at a pace that seems sufficiently comfortable that the runner is frequently tempted to accelerate in order to “bank some minutes” and get ahead of their time goal; resisting this temptation may be crucial for avoiding dramatic slowing late in the race (Humphrey, Hanson & Hanson, 2012; Magness, 2014). By contrast, in shorter races, such conservatism seems less important, apparently because immediate feelings of discomfort are generally sufficient to inform the runner that they should reduce their speed to avoid severe distress. Underscoring the unusual pacing demands in the marathon is that the mean percentage slowing in marathons (Deaner et al., 2015a) is far greater than documented in this 10 km race.

### Future work on decision making and pacing

Although the present study’s results, in conjunction with previous research, indicate that decision making contributes to the sex difference in pacing, additional research must be conducted to make strong tests of this hypothesis. Ideally, this work would investigate when and how runners establish their pre-race goals, including their target pace(s); runners’ estimates of the benefits, costs, and likelihoods of various race outcomes (e.g., dramatic slowing, setting a personal best, winning age group); runners’ psychological and physiological experiences during the race as they adjust their pace and modify their goals; and how these factors vary according to sex, age, experience, risk taking, training, ability, coaching, course difficulty, and race distance. Besides allowing a strong test of whether decision making contributes to the sex difference in pacing, such research may reveal unexpected and interesting interactions among physiological and psychological factors.

If it turns out that the sex difference in pacing partly reflects a sex difference in some aspect of decision making (e.g., over-confidence, risk perception, willingness to tolerate discomfort), then coaches and athletes may benefit by considering individual and group variation in these characteristics when planning training and racing. For example, training sessions aiming to improve pacing for males (or more confident females) may need to emphasize being conservative whereas corresponding sessions for females (or less confident males) may require encouraging more ambitious pacing.

### Limitations

The current study has several limitations. First, we evaluated pacing by comparing the pace of the first 3 miles to the final 3.2 miles and by comparing the pace of the first mile relative to the final 5.2 miles. Although these two pacing measures produced roughly similar outcomes with respect to sex differences among faster runners, other pacing measures could be explored and might yield different results. Second, although we succeeded in identifying sex, finishing time, and age as contributors to pacing variation, other factors must also be important. For example, in the study of 14 US marathons, greater race experience was associated with lesser slowing, although the effect was modest and did not eliminate the sex difference in pacing (Deaner et al., 2015a). As noted above, other relevant factors might include risk taking, training, and coaching. Third, although our sample size was large in terms of runners, the sample was drawn entirely from six years of the same race, the Bolder Boulder 10 km road race. This may limit the generalizability of our conclusions. Finally, many runners participated in more than one year of the race, and we did not cluster performances by runner. This could have led to *p*-values being smaller than they would have been if clustering was modeled explicitly.

## Conclusions

This study demonstrates that the sex difference in pacing that has been documented in marathons also occurs in a large 10 km road race, although the magnitude of the sex difference was smaller and limited to relatively fast runners, roughly men who finished in less than 53 min and women who finished in less than 60 min. This sex difference was robust in several respects, including that it occurred with two measures of pacing, with continuous and categorical specifications of the pacing measures, and when age was statistically controlled. These results suggest that the sex difference in pacing partly reflects a sex difference in some aspect of decision making. To make a strong test of this hypothesis, future research must directly address runners’ decision making and relate it to their pacing.

## Supplemental Information

10.7717/peerj.2235/supp-1 Data S1 Raw Data (2008–2013)

## Additional Information and Declarations

### Data Availability

The following information was supplied regarding data availability: The raw data has been supplied as Data S1.
